# Supplementary material for: A Systematic Study on Impact of Binder Formulation on Green Body Strength of Vat-Photopolymerisation 3D Printed Silica Ceramics Used in Investment Casting
Source: Polymers (Basel). 2023 Jul 24;15(14):3141. doi: 10.3390/polym15143141 (PMC10383664; doi:10.3390/polym15143141)
Supplement: Supplementary file 1 [file polymers-15-03141-s001.zip › polymers-2421376-supplementary.pdf]

Type of the Paper (Article)

Supplementary

Cure depth measurements and identification of printing parameters

It is critical to understand the optimum exposure energy for a binder mixture to cure the required layer thickness (100 μm) and therefore understand the characteristic reactivity of each binder mixture when illuminated by 405 nm UV light [1]. Jacob's working curve equation (Beer-Lambert Law) [2] is used to understand the key parameters like penetration depth and critical energy using the Cd and exposure energy. The Jacob's Working curve equation is as follows, **Eq. (S1)**.

$$C_d = D_p \times ln\left[\frac{E_0}{E_c}\right] \quad \textbf{(S1)}$$

where D_p is the Penetration Depth of the binder, E_0 is the initial light energy of the UV light source, and Ec is the critical exposure energy [3].

The Jacobs working curve represents ln (E_0) with the measured C_d value. E_0 is a calculated entity defined as a function of the product of the power of the light source and the amount of time the binder was exposed. For this study, the power of the light source is found to be 0.445 $mWcm^{-2}$ for the printer used, and the C_d is recorded for each binder for the exposure time from 8s to 20s. E_c and D_p are the functions of the y-intercept and slope of the straight line that fits well into the points plotted in the graph between C_d and E_0. Error! Reference source not found. is an example of the working curve of a binder used in the study).

In the straight-line equation y=ax-b, a is the slope of the plot, which is D_p, and b is the opposite of the y-intercept. Therefore, Ec can be calculated by substituting **Eq. (S1)**. Into the line equation, which gives E_c = exp (b/D_p). This method helps determine the E_c and D_p for all the binder mixtures. The D_p and E_c can be utilised to calculate the curing time required for a specific layer thickness for each binder mixture. However, if they have the same cure thickness, this method cannot differentiate between high and low cross-linked cured samples (i.e., semi-solid and solid cured samples). To overcome this phenomenon, the exposure time is increased by 30% of the calculated exposure time and rounded to the nearest whole number to ensure that the cured binder is fully solid with high cross-linking, showcasing good mechanical properties [3,4].

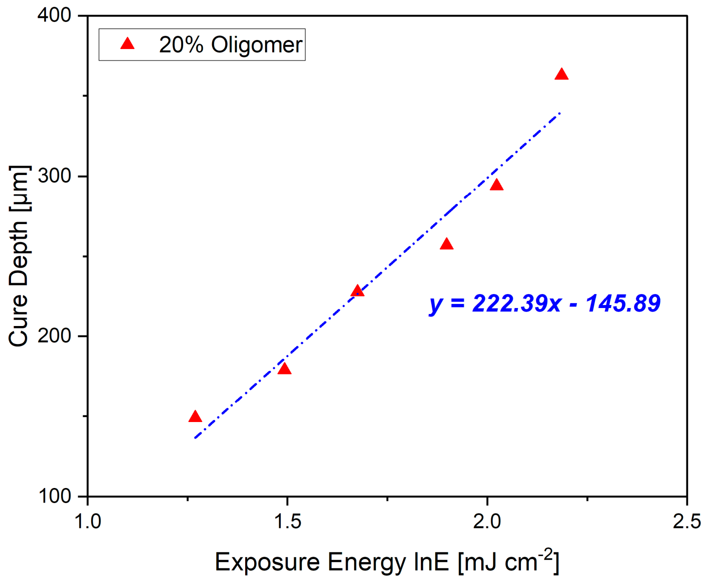

Figure S1 An example of a working curve for the binder with 20% UA with fitted linear line and its line equation to identify critical exposure energy and penetration depth.

References

1. Ozkan, B.; Sameni, F.; Karmel, S.; Engstrøm, D.S.; Sabet, E. Binder Stabilization and Rheology Optimization for Vat-Photopolymerization 3D Printing of Silica-Based Ceramic Mixtures. *J Eur Ceram Soc* **2023**, *43*, 1649–1662, doi:10.1016/j.jeurceramsoc.2022.11.032.
2. Halloran, J.W. Ceramic Stereolithography: Additive Manufacturing for Ceramics by Photopolymerization. *Annu Rev Mater Res* 2016, *46*, 19–40.
3. Oezkan, B.; Sameni, F.; Karmel, S.; Engstrøm, D.S.; Sabet, E. A Systematic Study of Vat-Polymerization Binders with Potential Use in the Ceramic Suspension 3D Printing. *Addit Manuf* **2021**, 102225, doi:https://doi.org/10.1016/j.addma.2021.102225.
4. Ozkan, B.; Sameni, F.; Bianchi, F.; Zarezadeh, H.; Karmel, S.; Engstrøm, D.S.; Sabet, E. 3D Printing Ceramic Cores for Investment Casting of Turbine Blades, Using LCD Screen Printers: The Mixture Design and Characterisation. *J Eur Ceram Soc* **2021**, doi:https://doi.org/10.1016/j.jeurceramsoc.2021.10.043.
